# Different weightings of input components to hippocampal CA1 place cells in young and aged rats

**DOI:** 10.1186/1471-2202-16-S1-P10

**Published:** 2015-12-18

**Authors:** Frances S Chance, Andrew P Maurer, Sara N Burke, Carol A Barnes

**Affiliations:** 1 Department of Data Driven and Neural Computing, Sandia National Laboratories, Albuquerque, NM 87123, USA; 2 Department of Neuroscience, University of Florida, Gainesville FL 32611, USA; 3 Evelyn F. McKnight Brain Institute, University of Arizona, Tucson, AZ 85721, USA; 4 ARL Div. of Neural Systems, Memory & Aging, University of Arizona, Tucson, AZ 85721, USA; 5 Departments of Psychology, Neurology and Neuroscience, University of Arizona, Tucson, AZ 85721, USA

## 

Hippocampal place cells vary both their firing rate and also the timing of their action potentials relative to the theta rhythm as an animal moves through space, suggesting that these neurons utilize both these features to encode spatial information. Place cells within the CA1 subfield receive synaptic input from CA3 (via the Schaffer collateral pathway) and also EC3 (layer 3 of entorhinal cortex via the perforant pathway). These pathways are thought to carry different modalities of information, for example auto-associative memories from CA3 and more sensory-driven information from entorhinal cortex. We seek to understand how both modalities of information are represented in the spiking outputs of CA1 place cells.

One model of CA1 phase precession [1] has proposed that these two input components drive CA1 spiking over spatially-offset but overlapping ranges of positions, and also over different ranges of theta phases. Because CA3 and EC3 inputs drive spiking at different theta phases in this model, changes in relative input strengths can be quantified by examining spike theta-phase distributions. For example, when one input component dominates CA1 spiking, the result is a unimodal theta-phase distribution, but if both input components drive CA1 spiking, their different theta-phase ranges produce a more bimodal theta-phase distribution.

In this study, we examine the spike patterns of CA1 place cells recorded from young and aged rats. Aged rat brains show a decreased number of functional synapses from CA3 to CA1 pyramidal cells compared to brains from younger rats (for review, see [2,3]). This suggests that theta-phase distributions of CA1 place-cell spiking will change with aging. We find that place cells from aged rats are more strongly theta-modulated, suggesting that CA3 input plays an important role in either driving or facilitating a second spiking component of CA1 activity.
